# Unexpected 3+ valence of iron in FeO_2_, a geologically important material lying “in between” oxides and peroxides

**DOI:** 10.1038/s41598-017-13312-4

**Published:** 2017-10-11

**Authors:** Sergey S. Streltsov, Alexey O. Shorikov, Sergey L. Skornyakov, Alexander I. Poteryaev, Daniel I. Khomskii

**Affiliations:** 1 M.N. Miheev Institute of Metal Physics of Ural Branch of Russian Academy of Sciences, Ekaterinburg, Russia; 2 Ural Federal University named after the first President of Russia B.N. Yeltsin, Theoretical Physics and Applied Mathematics Department, Ekaterinburg, Russia; 3 II. Physikalisches Institut, Universitat zu Koln, Koln, Germany

## Abstract

Recent discovery of the pyrite FeO_2_, which can be an important ingredient of the Earth’s lower mantle and which in particular may serve as an extra source of water in the Earth’s interior, opens new perspectives for geophysics and geochemistry, but this is also an extremely interesting material from physical point of view. We found that in contrast to naive expectations Fe is nearly 3+ in this material, which strongly affects its magnetic properties and makes it qualitatively different from well known sulfide analogue - FeS_2_. Doping, which is most likely to occur in the Earth’s mantle, makes FeO_2_ much more magnetic. In addition we show that unique electronic structure places FeO_2_ “in between” the usual dioxides and peroxides making this system interesting both for physics and solid state chemistry.

## Introduction

Recent discovery of a new iron oxide FeO_2_, which does not exist at normal conditions, but can be stabilized at a very high pressure (76 GPa) and temperature (1800 K)^1^ may dramatically shift our understanding of how Earth is formed and what was a source of oxygen and water in interior of our planet. FeO_2_ is expected to appear in the Earth’s lower mantle below 1800 km, according to^1^ in the pyrite structure, and start to dominate over other Fe oxides at higher pressures. The composition of the mantle is extremely important for the seismology, since it determines convection processes. There were proposed a number of structural models based on different ratio of ferropericlase (a solid solution of FeO and MgO), bridgmanite (Mg,Fe,Al)(Al,Fe,Si)O_3_, (Mg,Fe)_2_SiO_4_ olivine and other compounds^2–5^, but none of them took into account the existence of FeO_2_. Moreover, physical properties of this material are completely unexplored. One might expect that they can be highly unusual, since on one hand Fe ion in FeO_2_ formally should have exceptionally high oxidation state, 4+. Since the O-O distance in FeO_2_ is 1.89 Å it is not likely that there can be a strong bonding between the O ions, like in molecular oxygen (where the O-O bond distance is 1.21 Å) and one may indeed expect that Fe ions will adopt 4+ valence state and then FeO_2_ is in a negative charge transfer regime^6–9^. This may result in self-doping^10^ and also to bond or charge disproportionation^11,12^, inversion of the crystal field splitting^13^ or nontrivial magnetic structures. On the other hand, the presence of the ligand-ligand dimers may also strongly affect physical properties of FeO_2_ as it does in the actual pyrite FeS_2_ (“the fool’s gold”). However, O-O distance in FeO_2_ is 1.89 Å, much larger than in molecular oxygen (1.21 Å) or (O_2_)^2−^ ion as in the usual peroxides like BaO_2_, MgO_2_ (1.49 Å).

**Figure 1:**
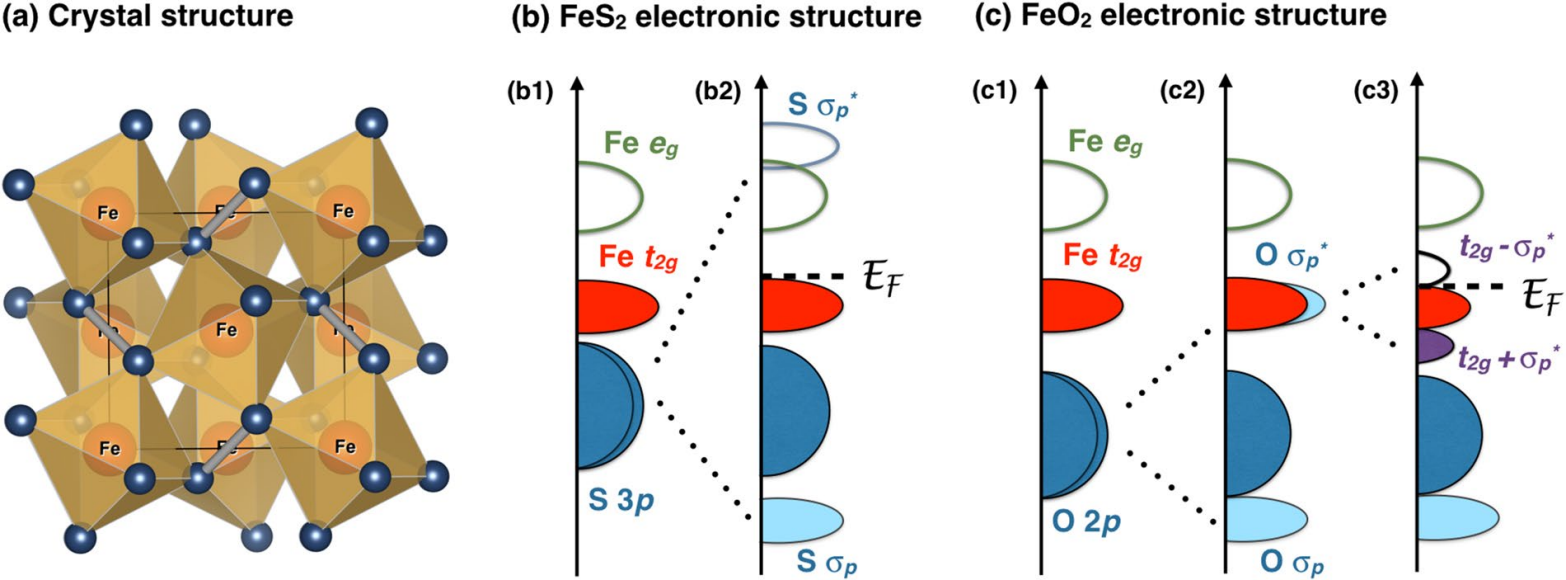
(**a**) Crystal structure of FeO_2_ and FeS_2_ can be visualized as a rocksalt structure like FeO with O ions replaced by S_2_ (in FeS_2_) or O_2_ (in FeO_2_) dimers. Fe ions are yellow, while O (or S) ions, forming dimers, are shown in blue. (**b**) and (**c**) Schematic band structure of FeS_2_ and FeO_2_.

Iron peroxide was found to have the same pyrite crystal structure as FeS_2_^1^, see Fig. 1a, and there is not much difference between oxygen and sulfur from chemical point of view. Thus, it is tempting to consider FeO_2_ as a complete analogue of FeS_2_^14^. Since FeS_2_ is known to be a diamagnetic insulator with Fe ions adopting 2+ valence state^15–17^, one might expect that the same is true for FeO_2_. The first indication that such a picture is oversimplified follows from the recent theoretical study^14^, where FeO_2_ was found to be metallic at the pressures where it does exist.

In the present paper we describe electronic and magnetic properties of FeO_2_. We show that FeO_2_ is completely different from FeS_2_, and so are the physical properties of these compounds. The oxidation state of Fe ion in FeO_2_ is not 2+, as in FeS_2_, but close to 3+. This strongly affects magnetic properties of FeO_2_, since having 3*d* ^5^ electronic configuration, Fe^3+^ ions may have a magnetic moment. Our comprehensive theoretical calculations using combination of the density functional and dynamical mean-field theories (DFT + DMFT) demonstrate that there is indeed a highly nontrivial temperature dependence of the magnetic susceptibility in FeO_2_. We found out that the origin of the difference in magnetic properties between FeO_2_and FeS_2_ and of the metallic character of FeO_2_ is a much smaller bonding-antibonding splitting for ligand σ orbitals in the peroxide dimer O_2_ as compared with S_2_, and a total shift of oxygen 2*p* levels relative to 3*p* levels of sulfur. This feature of the electronic structure is rather general and important for other dioxides, which can exist in Earth’s mantle or in inner parts of exoplanets.

We start with FeS_2_, electronic and magnetic properties of which are well understood. As discussed above, one might naively expect to have Fe^4+^ ions with 3*d* ^4^ electronic configuration in FeS_2_, since usually sulfur has a valence 2-. This would shift Fe 3*d* band very low in energy, below S 3*p*, and would result in a self-doping and a metallic conductivity^7^, which strongly disagrees with the experimental fact that FeS_2_ is a semiconductor^16,17^.

The explanation of this contradiction lies in the specific features of its crystal structure, namely the presence of the S_2_ dimers. There are sulfurs 3*p* orbitals, which are directed exactly to each other in these dimers. They form such a strong bond that the antibonding ${{\sigma }}_{p}^{\ast }$ orbitals turn out to be higher in energy than the Fe *e*_*g*_ orbitals, see Fig. 1(b2). This leads to a formal valency of sulfur “1-”, (or to (S_2_)^2−^ dimers), and Fe ions become 2+ with the 3*d* ^6^ electronic configuration. Fe ions are in the ligand octahedra in pyrite structure. Strong crystal field splitting between the *t*_2*g*_ and *e*_*g*_ bands (~3.5 eV in case of FeS_2_, see Supplemental materials - SM^18^) counteracts the Hund’s rule and stabilizes the low spin configuration with all six 3*d* electrons occupying *t*_2*g*_ sub-shell. This makes FeS_2_ diamagnetic and insulating^19^.

The electronic structure of FeO_2_ is rather different from a sulfide counterpart. We sketched how this difference appears in Fig. 1c (while the results of the actual calculations performed within generalized gradient approximation, GGA, as well as the details of such calculations are presented in Fig. S1 in SM^18^), starting from the hypothetical FeO_2_ having FCC lattice (like NaCl), where O ions do not form dimers and where there are basically three bands O *p*, Fe *t*_2*g*_, and Fe *e*_*g*_, see Fig. 1(c1).

**Figure 2:**
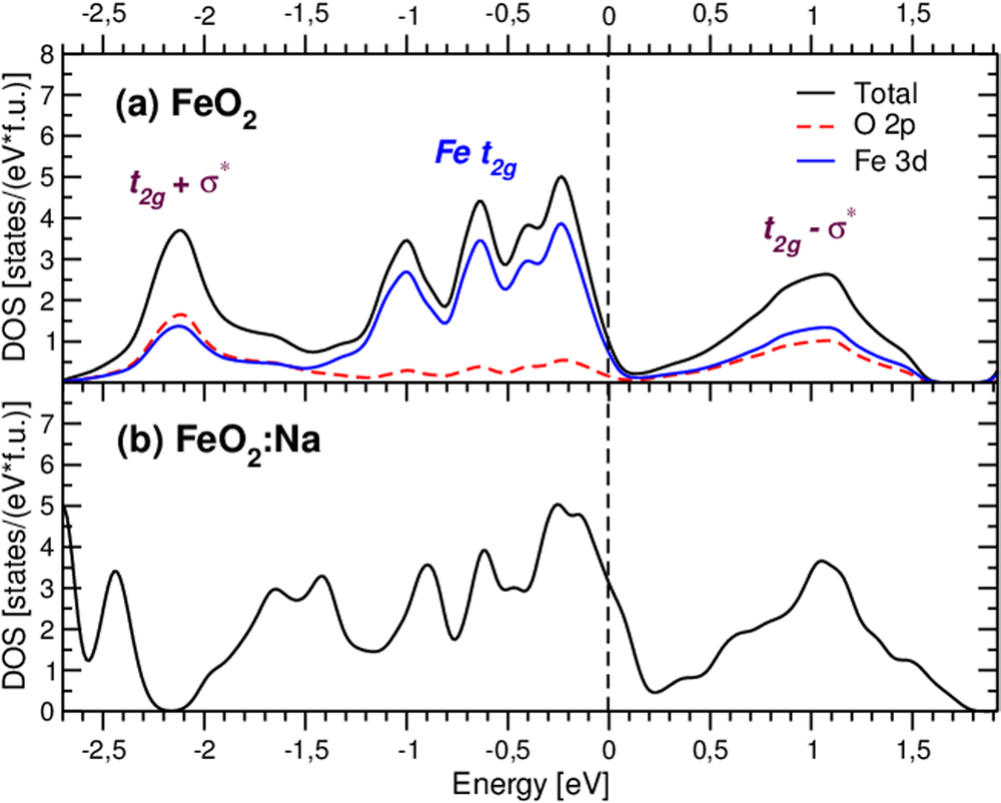
Total and partial density of states (DOS) in the nonmagnetic GGA calculations (**a**) for FeO_2_ and (**b**) FeO_2_ doped by Na (25%). Fermi energy is in zero.

First of all, as follows from our GGA calculations, the oxygen 2*p* levels are shifted down relative to the Fe 3*d* states, as compared with the 3*p* levels of sulfur. Besides, as was mentioned above, the presence of the ligand-ligand dimers in real FeO_2_ results in bonding-antibonding splitting, but since oxygen 2*p* orbitals are much less extended than sulfur 3*p* orbitals, this bonding-antibonding splitting in the O_2_ dimer is expected to be much smaller. As a result the antibonding O ${{\sigma }}_{p}^{\ast }$ orbital appears not above *e*_*g*_ (like in FeS_2_), but exactly in the place, where Fe *t*_2*g*_ bands lie, see Fig. 1(c2). Then, first of all, part of the Fe *t*_2*g*_ electrons would be transferred to oxygens, shifting Fe valence in the direction of 3+. Second, the hybridization between Fe 3*d* and O ${{\sigma }}_{p}^{\ast }$ orbitals again makes bonding and antibonding combinations, which are labeled as ${t}_{2g}+{{\sigma }}_{p}^{\ast }$ and ${t}_{2g}-{{\sigma }}_{p}^{\ast }$ in Fig. 1(c3) respectively. The density of states (DOS) plot in the vicinity of the Fermi energy as obtained in conventional GGA is presented in Fig. 2(a). These ${t}_{2g}+{{\sigma }}_{p}^{\ast }$ and ${t}_{2g}-{{\sigma }}_{p}^{\ast }$ bands are centered at −2 and 1 eV in Fig. 2(a). Note that these bands have nearly the same contributions from Fe *t*_2*g*_ and O 2*p* (${{\sigma }}_{p}^{\ast }$) states. Moreover, it is clear that peaks below and above the Fermi level are not bonding and antibonding, since there is no contribution from O 2*p* band below *E*_*F*_. These are nonbonding and antibonding states.

This salient feature of FeO_2_, that the antibonding *σ* ^*^ orbital falls exactly into the Fe *t*_2*g*_ band, determines the main physical properties of FeO_2_, which are very different from FeS_2_, see Table 1. First of all, since there appear additional bands at the Fermi level, while the number of electrons is the same, FeO_2_ is not a band insulator (as FeS_2_), but a metal.

**Table 1:** Comparison of different physical properties of FeS_2_ and FeO_2_, as follows from the DFT and DFT + DMFT calculations.

	Fe valence	Electric properties	Magnetic properties
FeS_2_	2+	insulator	diamagnetic
FeO_2_	3+	metal	paramagnetic

There are eight *t*_2*g*_ bands, each doubly degenerate with respect to spin, below the Fermi energy for the unit cell consisting of four formula units (f.u.), which are occupied by 4 electrons per each Fe ion (Fig. S1 in SM^18^). In addition there are four bonding ${t}_{2g}+{{\sigma }}_{p}^{\ast }$ bands with nearly 50% contribution of the Fe *t*_2*g*_ states (see partial DOS presented in Fig. 2), which adds approximately one more electron to each Fe ions. As a result Fe ions in FeO_2_ are nearly 3+ with 3*d* ^5^ electronic configuration, while in FeS_2_ they are 2+.

In contrast to Fe^2+^, which is nonmagnetic with ${t}_{2g}^{6}$ configuration at large pressure, Fe^3+^ ion even in the low-spin state has a magnetic moment. Moreover, the oxygen *σ* ^*^ states are half-filled in FeO_2_, and thus they can also contribute to the total magnetic moment.

Second, the Fermi level appears to be in a very specific position. On one hand it is almost in the pseudogap, so that the Stoner criterion for ferromagnetism (FM) is formally not fulfilled, and this is the reason why magnetic solutions does not survive in the GGA (we also checked stability of magnetic solutions at other *q*-vectors, corresponding to AFM-I and AFM-II magnetic structure of FCC lattice of Fe ions^20^). On the other hand it is just on the border line between bands corresponding to localized *t*_2*g*_ electrons and antibonding molecular ${t}_{2p}-{{\sigma }}_{p}^{\ast }$ states. This is very important for magnetic properties of stoichiometric and non-stoichiometric FeO_2_ as we will show latter.

While conventional DFT is exceptionally useful for understanding of the basics of the electronic structure in FeO_2_, it does not take into account strong Coulomb correlations, which are known to be important for description of the physical properties of many transition metal compounds. We treated correlation effects using the DFT + DMFT method^21^. Hubbard *U* was calculated to be 6 eV, ${J}_{H}=0.9$ eV, other details can be found in SM^18^.

Correlation effects manifest themselves basically via the renormalization of the GGA DOS near the Fermi level, ${m}^{\ast }/m$~1.2–1.6 (depending on the orbital), resulting spectral functions are shown in Fig. S2 of SM. FeO_2_ is a bad metal for experimental pressure of 76 GPa. There are 4.8 electrons in the *t*_2*g*_ shell, which certifies that Fe is 3+ in FeO_2_. The local magnetic moment $\langle \sqrt{{m}_{z}^{2}}\rangle$ was found be 1.5 ${\mu }_{B}$. The contribution from the *t*_2*g*_ orbitals to the total local magnetic moment, ${\langle {m}_{z}^{2}\rangle }_{{t}_{2g}}=1.08{\mu }_{B}^{2}$, exactly corresponds to the low spin state of 3*d* ^5^ configuration. There is, however, also an additional contribution, ${\langle {m}_{z}^{2}\rangle }_{{e}_{g}}=1.04{\mu }_{B}^{2}$, due to a partial polarization of the ligand electrons residing *e*_*g*_ shell of transition metal (see detailed discussion in Supplemental materials). In spite of the fact that there are magnetic moments on Fe ions, they do not order, so that FeO_2_ stays paramagnetic down to 190 K (we checked FM and AFM-I). Even lower temperatures can be reached in our calculations by using a truncated Hamiltonian, which includes only Fe *t*_2*g*_ and O 2*p* states (this choice of impurity orbitals gives the same spectral functions in vicinity of the Fermi level and very similar $\chi (T)$ as full 3*d* Hamiltonian). In this case we were able to go down to 60 K, and again FeO_2_ does not order in our calculations even at these temperatures. This may seem somewhat surprising since having a rather large bandwidth (and hence hopping parameters) one might expect large superexchange interaction between Fe ions, if spins would have been localized.

**Figure 3:**
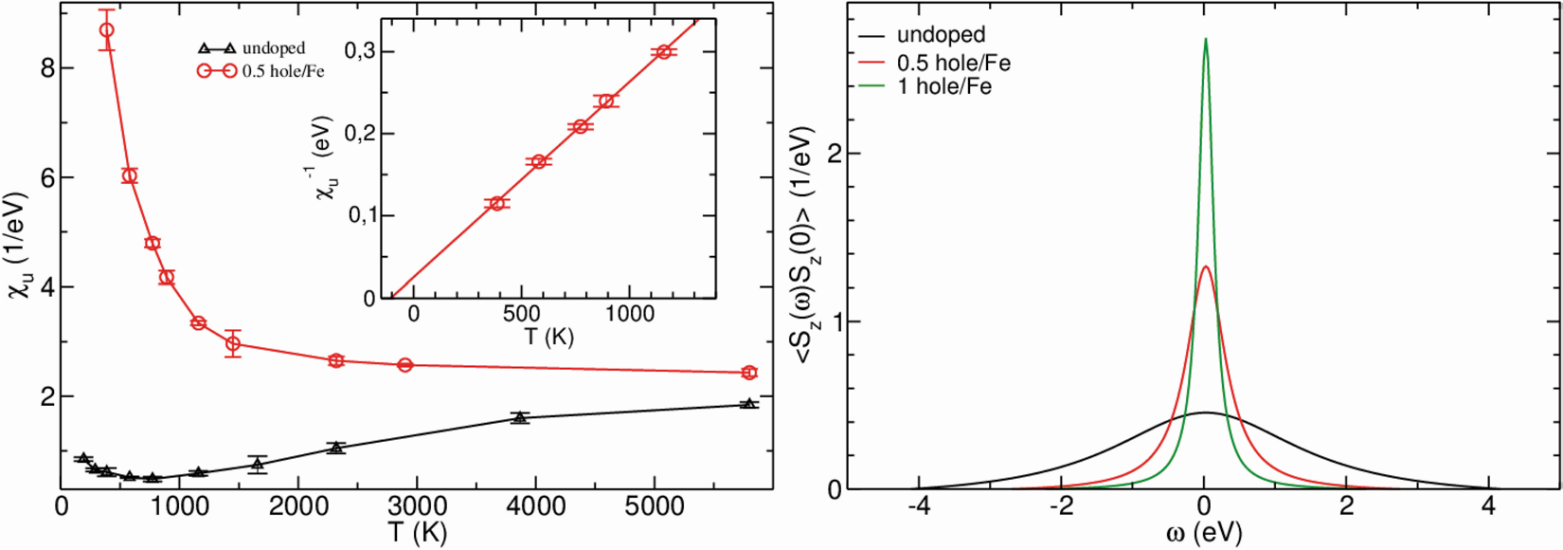
Results of the DFT + DMFT calculations. Left panel: uniform magnetic susceptibility for pure FeO_2_ and $\frac{1}{2}$ hole/Fe doping (red circles). Inset shows magnetic susceptibility for 0.5 hole per Fe atom as a function of 1/T. Right panel shows local magnetic susceptibility as a function of frequency for different doping.

In order to estimate the degree of the spin localization we calculated the analytical continuation on real frequency of the spin-spin correlator $\langle {S}_{z}(i\omega ){S}_{z}(o)\rangle ={\int }_{0}^{\mathrm{1/}{k}_{B}T}d\tau \langle {S}^{z}(\tau ){S}^{z}(o)\rangle {e}^{i{\omega }_{n}\tau }$, where $\tau$ is an imaginary time, see right panel in Fig. 3 ^22,23^. The width of this correlator is inversely proportional to the lifetime of a magnetic moment. For example in a pure metallic iron, where ${t}_{2g}-{e}_{g}$ crystal field splitting is small, iron ion is in a high-spin state. The magnetic moment can be considered to be localized, with the full width at half maximum (FWHM) of about 0.2 eV for the less localized $\gamma$-Fe and 0.1 eV for the more localized $\alpha$-Fe^22,24^. From the inset of Fig. 3 one may see that in FeO_2_ FWHM of the spin-spin correlator is ~3 eV, which demonstrates that the magnetic moments can hardly be considered as localized.

In DMFT one can calculate the uniform magnetic susceptibility ${\chi }_{u}(T)$ as a response to an external magnetic field, which is introduced via Zeeman splitting $\delta E$ in the Hamiltonian:

1 $${\chi }_{u}(T)=\frac{\delta m}{\delta H}=\frac{{n}^{\uparrow }-{n}^{\downarrow }}{\delta E}{\mu }_{B}^{2}.$$

Here *m* is the magnetization, ${n}^{\uparrow }$ and ${n}^{\downarrow }$ are total occupations for spin up and down. This direct calculation of the uniform magnetic susceptibility, ${\chi }_{u}(T),$ shows that it has a nontrivial temperature dependence. Namely, with increasing temperature ${\chi }_{u}$ first decreases (for $T < {T}^{\ast }\,=$ 750 K), and then starts to increase almost linearly above ${T}^{\ast }$, which resembles the behaviour of the pnictides^25^. Detailed analysis of these data^18^ shows that such an unusual for 3D system behavior is due to a specific position of the Fermi level in between the localized *t*_2*g*_ and antibonding ${t}_{2g}-{\sigma }_{p}^{\ast }$ states. At low temperature the particle-hole excitations occur within the localized Fe *t*_2*g*_ states and ${\chi }_{u}(T)$ goes down with temperature, resembling the Curie-Weiss law. Increasing temperature further ($T > {T}^{\ast }$ K), we start to excite molecular-like ${t}_{2g}-{\sigma }_{p}^{\ast }$ states, which leads to a completely different temperature dependence.

This means that the electron and hole doping, which is likely to occur in Earth’s mantle, would result in a very different temperature dependences of magnetic susceptibility, since we shift the Fermi level to the peaks corresponding to *qualitatively* different states (localized and molecular-like). There are many different elements besides Fe (5.8%) and O (44.8%) in the Earth’s mantle, and one may expect that Mg (~22.8%), Si (~21.5%), Ca (~2.3%) or Na (0.3%)^26^ may dope FeO_2_ and change its properties dramatically. Indeed, the Fermi level in stoichiometric FeO_2_ is on the steep slope of a large peak in DOS, and changing its position we strongly affect both magnetic and electronic properties.

The electron doping will shift the Fermi level to antibonding molecular-like ${t}_{2g}-{\sigma }_{p}^{\ast }$ states, which is unlikely to provide a large magnetic response in the simplest rigid-band shift model. Moreover, by doing this we transform Fe ion into the nonmagnetic low-spin $3{d}^{6}$ configuration, corresponding to the 2+ oxidation state, so that only a small electron doping can increase magnetic moment. In addition the electron doping is rather unfavourable from structural point of view: the population of the strongly antibonding ${t}_{2g}-{\sigma }_{p}^{\ast }$ orbital would significantly weaken O_2_ dimers, existing in the pyrite structure. Thus, at first sight the hole doping is expected to be much more effective for making FeO_2_ magnetic: the Fermi level would then be shifted to the large peak corresponding to localized Fe *t*_2*g*_ electrons.

**Figure 4:**
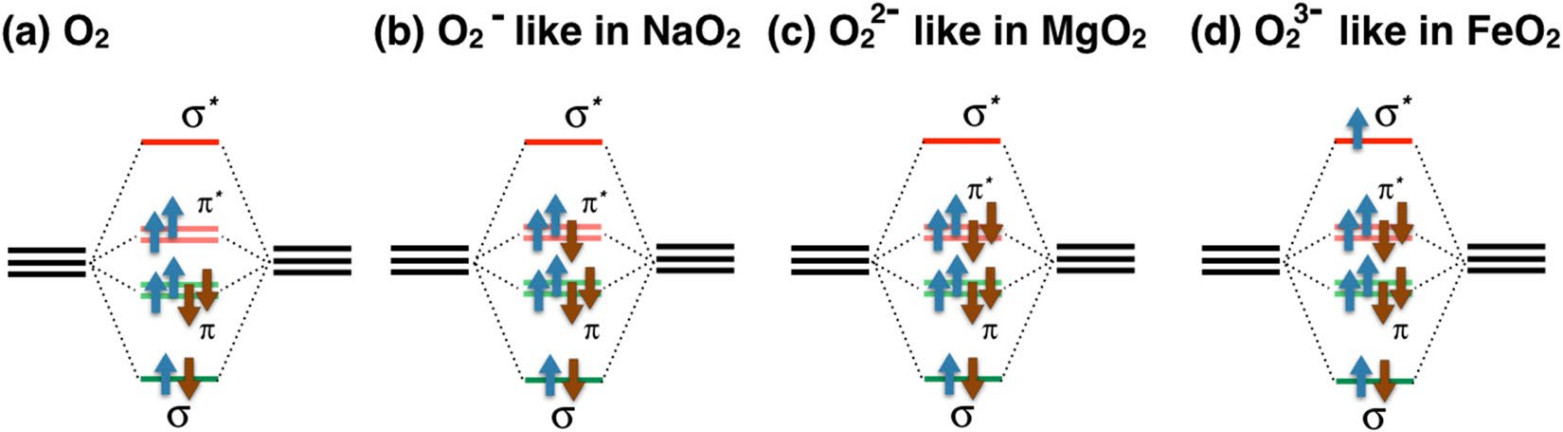
Occupation of oxygen 2*p* orbitals in different compounds with O_2_ dimers. The system gains (loses) energy by occupation of green bonding (red antibonding) bonds.

We checked different types of hole and electron dopings by the GGA calculations (for ferromagnetic order) performing full structural optimization, starting from the pyrite structure and substituting 25% of Fe by different ions such ions as Mg, Si, and Na. Mg doping formally changes valence of the peroxide O_2_ group from 3- in FeO_2_ to 2- in MgO_2_ (see Fig. 4(c) and (d)), but it has no influence either on band structure or on magnetic properties of the system: unoccupied *σ* ^*^ band corresponding to the Mg(O_2_)^2−^ unit appears just above the Fermi level and does not provide any holes to the Fe ions. In NaO_2_ superoxide the O_2_ “molecule” is 1-, see Fig. 4(b) and also ref.^27^, and hence by Na we depopulate O ${\pi }^{\ast }$ bond, which will be immediately refilled by the Fe *t*_2*g*_ electrons. This leads to the shift of the Fermi level downwards, see Fig. 2b, and results in the magnetic instability. In the GGA calculations the magnetic moments on Fe ions were found to be ~0.4 ${\mu }_{B}$. Si doping keeps FeO_2_:Si nonmagnetic, but only in unrelaxed crystal structure. After lattice optimization there appears two very different O_2_ dimers, which help to form magnetic moment ~0.4 ${\mu }_{B}$ even in the case of the light electron doping. But the most effective are Fe vacancies (25%), which give magnetic ground state in the GGA calculations with magnetic moments ~0.6 ${\mu }_{B}$.

Thus, we see that there are plenty of possibilities for FeO_2_ to be magnetic due to different types of doping or because of non-stoichiometry. It is hard to expect, however, that FeO_2_ would order magnetically in the Earth’s mantle, because of very high temperatures, ~1000–2000 K, but even in a paramagnetic state it may still provide local magnetic moments. The direct DFT + DMFT calculations within the rigid-band shift approximation (as one can see from Fig. 2b, the band structure does not change dramatically with doping) show the drastic increase of the uniform magnetic susceptibility with hole doping, see Fig. 3. it is now Curie-Weiss like in a wide temperature range and the spin-spin correlation function demonstrates an increase of the local magnetic moments lifetime (i.e. decrease of the width of the correlator, see inset in Fig. 3) with doping.

In addition to a possible importance of our findings for geoscience, FeO_2_ represents an exceptional interest also for physics and solid state chemistry, since it lies on the borderline between the stable dioxides of transition metals, such as TiO_2_ VO_2_, CrO_2_ etc., and equally stable oxides and sulfides having pyrite structure, such as NaO_2_, KO_2_, FeS_2_ etc. FeO_2_ may thus be considered as a “bridge” between dioxides and peroxides/disulfides, and it displays properties of both.

There is a well known concept in physics, introduced by Zaanen, Sawatzky and Allen^9^, that going along a row in the periodic table from the left to the right, or increasing valence of a metal in a transition metal oxide, we go over from the Mott insulator, where the band gap is defined by Hubbard *U*, to a charge-transfer regime, where it is given by the charge transfer (CT) from a ligand to a metal, ${{\rm{\Delta }}}_{CT} > 0$, and finally to the state, where ${{\rm{\Delta }}}_{CT}$ becomes negative with ligands donating some of their electrons to a metal (so called self-doping)^8,9^.

In peroxides the situation with the CT energy is “inverted” from the beginning: as we have seen, in FeS_2_ part of electrons are transferred from sulfur to Fe. CoS_2_, NiS_2_, MgO_2_, KO_2_ and many other materials are just the same: ligand *σ* ^*^ and sometimes even ${\pi }^{\ast }$ orbitals donate (see Fig. 4) at least one electron for a metal, i.e. oxygen is 1- or even 1/2-. With FeO_2_ one returns to normal transition metal oxides, where oxygen’s valency is 2-, but there is still one step to make since O is 1.5- in FeO_2_. Thus, we see that FeO_2_ indeed lies “in between” oxides and peroxides/disulfides, which makes it an especially interesting material from physical point of view.

A simple qualitative difference between normal (di)oxides and peroxides is the following: On one hand, when the main “structural unit” in a system is a single O ion, like in dioxides of the type of TiO_2_, VO_2_, its “natural” state is O^2−^, and counting from that, we see that e.g. in FeO_2_ the CT energy would be negative, ${{\rm{\Delta }}}_{CT} < 0$, i.e. the electrons would be transferred from O^2−^ to Fe (as it happens already in CrO_2_^10^). But in peroxides, as well as, e.g., in FeS_2_, the natural “structural unit” is the O_2_ or S_2_ dimer. Such dimer can be in different charge states: neutral O_2_ molecule, Fig. 4a; (O_2_)^−^ molecular ion (say in NaO_2_, KO_2_), Fig. 4b; or (O_2_)^2−^ ion as in MgO_2_, Fig. 4c.

The more electrons we put on such a dimer, the more we fill antibonding states, which gradually destabilizes the very O_2_ dimers. But till (O_2_)^2−^ it is still reasonably harmless, we fill “weakly” antibonding states (${\pi }^{\ast }$), see Fig. 4. But as soon as one starts to occupy the upper *σ* ^*^ states, the very dimers start to become more and more destabilised, which we indeed see in FeO_2_: the O-O distance in (O_2_)^3−^ dimers is 1.89 Å^1^ - much larger than 1.49 Å for (O_2_)^2−^ dimer in MgO_2_^28^ or 1.32 Å for (O_2_)^−^ in NaO_2_^29^. Already MgO_2_, having 4 electrons on antibonding ${\pi }^{\ast }$ orbitals, see Fig. 4(c), readily decomposes at zero pressure^30^. In FeO_2_ we lose even more energy occupying antibonding *σ* ^*^ orbital, see Fig. 4(d). This makes FeO_2_ even less stable in the pyrite structure, than MgO_2_, so that it can be stabilised only at a very high pressure.

Summarising, we see that the recently discovered pyrite-like FeO_2_^1^ is even more exotic than it was initially thought. Unexpected valence states, nontrivial magnetic properties, stabilization of local magnetic moments by non-stoichiometry or doping by such abundant constituents of Earth’s mantle such as Si (and Na) and finally its special place between (di)oxides and peroxides make FeO_2_ extremely interesting not only for geoscience, but also for the condensed matter physics and solid state chemistry.

### Data availability statement

No datasets were generated or analysed during the current study.

## Electronic supplementary material

Supplementary information
