# Supplementary material for: Unexpected 3+ valence of iron in FeO2, a geologically important material lying “in between” oxides and peroxides
Source: Sci Rep. 2017 Oct 11;7:13005. doi: 10.1038/s41598-017-13312-4 (PMC5636914; doi:10.1038/s41598-017-13312-4)
Supplement: Supplementary file 1 — Supplementary information [file 41598_2017_13312_MOESM1_ESM.pdf]

Supplementary materials: Unexpected 3+ valence of iron in FeO_2, a geologically important material lying “in between” oxides and peroxides

Sergey S. Streltsov[1,2,*], Alexey O. Shorikov[1,2], Sergey L. Skornyakov[1,2], Alexander I. Poteryaev[1], and Daniel I. Khomskii[3]

[1]M.N. Miheev Institute of Metal Physics of Ural Branch of Russian Academy of Sciences, Ekaterinburg, Russia
[2]Ural Federal University, Theoretical Physics and Applied Mathematics Department, Ekaterinburg, Russia
[3]II. Physikalisches Institut, Universität zu Köln, Köln, Germany
[*]streltsov.s@gmail.com

ABSTRACT

Supplementary materials for “Unexpected 3+ valence of iron in FeO_2, a geologically important material lying “in between” oxides and peroxides”.

Details of the calculations

For *ab initio* calculations we used full-potential Wien2k[1] and pseudopotential Quantum ESPRESSO (QE)[2] codes. The cutoff energy for wave-functions in QE was chosen to be 45 Ry. The parameter of the plane-wave expansion in Wien2k calculations was set to be $R_{MT}K_{max}$ = 7, where R_{MT} is the smallest atomic sphere radii ($R^{O}_{MT} = 1.6$ a.u., $R^{Fe}_{MT} = 1.8$ a.u.) and K_{max} - plane wave cut-off. The crystal structures for the calculations were taken from Ref.[3,4]. The on-site Hubbard $U = 6$ eV and Hund’s intra-atomic exchange $J_H = 0.9$ eV were estimated in QE[5] on the same Wannier functions, which were used in construction of a small noninteracting Hamiltonian used in subsequent GGA+DMFT calculations[6,7]. Note, that these values agree well with results of previous calculations of U for other Fe oxides at high pressure[8]. It is shown in the main part of paper that Hubbard correlations have minor influence on the electronic properties of FeO_2. Therefore we have not investigated how they change with U.

We used segment CT-QMC method to solve an impurity problem[9]. The Hamiltonian includes Fe $3d$ and O $2p$ states and was constructed on a fine-grid $16 \times 16 \times 16$ mesh in $k-$space. The double counting correction was set to $E_{dc} = U(n_{DMFT} - \frac{1}{2})$[6], with n_{DMFT} the total number of $3d$ electrons self-consistently obtained within DMFT.

For the calculation we used the crystal structure presented in[4] (in the extended data tables 1 and 2). We chose the one corresponding to the powder data.

GGA results

In Fig. S1 results of the nonmagnetic GGA calculations for FeO_2 and FeS_2 are presented. One may see that FeS_2 is an insulator, while FeO_2 is metallic. We note in passing that the transition to insulating state found in Ref.[10] at larger volumes obviously corresponds to smaller pressures, below critical value $P_c = 76$ GPa, at which FeO_2 can be synthetized[4].

In the GGA calculations for defect and doped FeO_2 we allowed a relaxation of the crystal structure. Using total energy calculations we estimated the nearest neighbor exchange coupling, J, in the case of 25% Fe vacancies ($J = 20$ K) and simulated the uniform magnetic susceptibility in the classical Heisenberg model using QMC calculations as implemented in ALPS code[11] (see Fig. S4).

Dynamical electronic correlations (DFT+DMFT results) and magnetic properties of FeO_2

We used wannier function projection procedure[12] as realized in the Quantum ESPRESSO (QE)[2] code to extract noninteracting GGA hamiltonian H_{DFT}, which included both Fe $3d$ and O $2p$ states. Full many-body Hamiltonian to be solved by the GGA+DMFT is written in the form:

$$\hat{H} = \hat{H}_{DFT} - \hat{H}_{dc} + \frac{1}{2} \sum_{i,\alpha,\beta,\sigma,\sigma'} U^{\sigma\sigma'}_{\alpha\beta} \hat{n}^{d}_{i\alpha\sigma} \hat{n}^{d}_{i\beta\sigma'}. \quad (1)$$

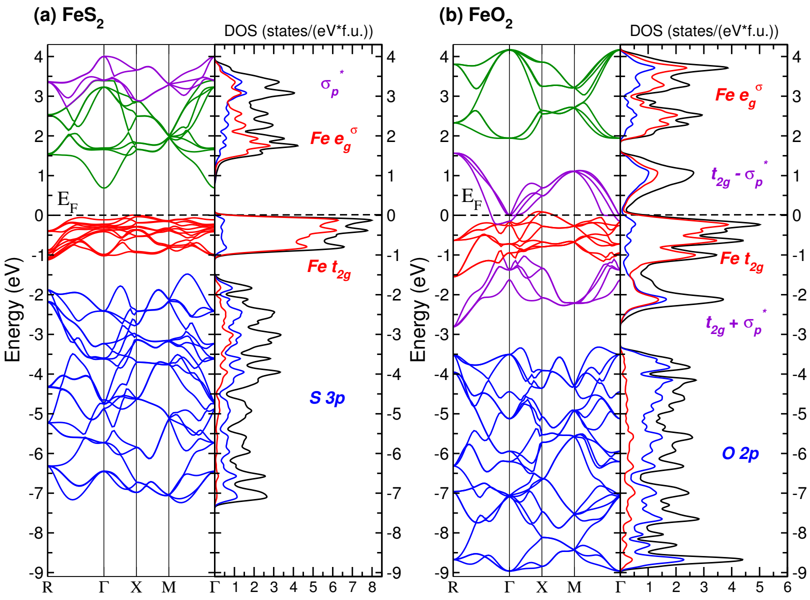

Figure S1. Results of the nonmagnetic GGA calculations (a) for FeS_2 and (b) for FeO_2. There are 4 formula units (f.u.) in the unit cell. Total density of state (DOS) is shown in black, Fe $3d$ states in red, and ligand $2p/3p$ in blue. Color coding in the band structure is used for illustration purpose only.

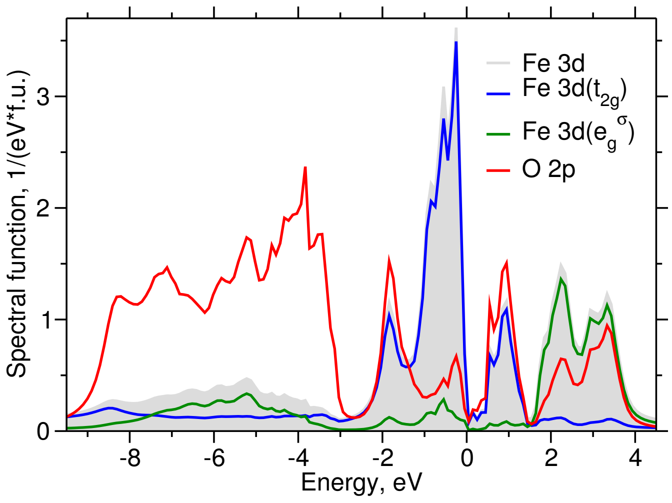

Figure S2. The DFT+DMFT spectral function calculated for β=20 eV^{-1}.

Here $U^{\sigma\sigma'}_{\alpha\beta}$ is the Coulomb interaction matrix, $\hat{n}^{d}_{i\alpha\sigma}$ is the occupation number operator for the d electrons with orbitals α or β and spin indexes σ or σ' on the i-th site. The term $\hat{H}_{dc}$ stands for the d-d interaction already accounted for in the DFT, so called double-counting correction, which was chosen to be $\hat{H}_{dc} = \bar{U}(n_{\mathrm{dmft}} - \frac{1}{2})\hat{I}$. Here n_{dmft} is the self-consistent total number of d electrons obtained within the DFT+DMFT, $\bar{U}$ is the average Coulomb parameter for the d shell.

The elements of $U^{\sigma\sigma'}_{\alpha\beta}$ matrix are parameterized by U and J_H according to procedure described in Ref.[13]. The effective impurity problem for the DMFT was solved by the hybridization expansion Continuous-Time Quantum Monte-Carlo method (CT-QMC)[9]. Spectral functions on real energies were calculated by Maximum Entropy Method (MEM)[14]. The values of Coulomb repulsion parameter U and Hund's exchange parameter J_H were found to be U = 6.0 eV and J_H=0.9 eV using constrained LDA calculations[5]. The calculations were performed with the AMULET code[7].

Spectral function for undoped FeO_2 calculated for inverse temperature $\beta = 1/T$=20eV^{-1} is shown in Fig. S2. One may see that the main effect of the electronic correlations is a renormalization of the spectra in the vicinity of the Fermi level, $m^*/m \sim$ 1.2-1.6 (depending on the orbital).

We start analysis of the magnetic properties of FeO_2 with discussion of the expectation value of squared magnetization $\langle m_z^2 \rangle$,

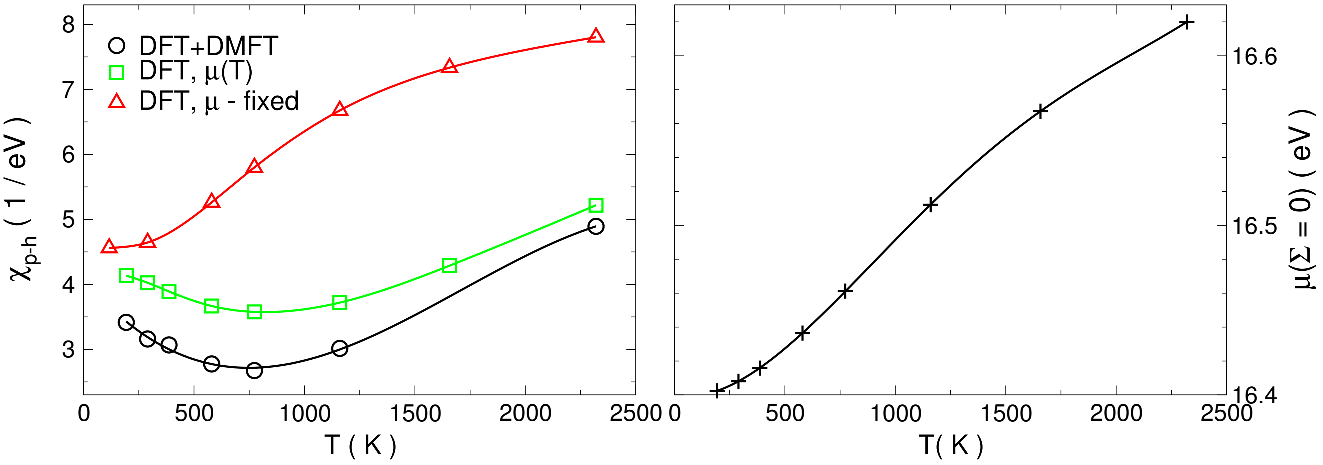

Figure S3. Left panel: Temperature dependence of the spin susceptibility as obtained employing the particle-hole bubble approximation. The symbols correspond to results obtained according to Eq. 2 with Green functions computed within DFT+DMFT (circles), and those in the non-interacting case with fixed (triangles) and temperature-dependent (squares) chemical potential. Right panel: Chemical potential in the non-interacting limit as a function of temperature.

which, e.g., for $\beta = 15$ eV^{-1} was found to be 2.32 μ_B^2. In DMFT it is calculated as $\langle m_z^2 \rangle = \langle \left(\sum_m n_m^{\uparrow} - n_m^{\downarrow} \right) \left(\sum_{m'} n_{m'}^{\uparrow} - n_{m'}^{\downarrow} \right) \rangle$, where n_m are the occupancies of corresponding orbitals. Thus, there will be three contributions to $\langle m_z^2 \rangle$, which come from the t_{2g} orbitals, the e_g orbitals, and mixed t_{2g}/e_g terms: $\langle m_z^2 \rangle_{t_{2g}} = 1.08\mu_B^2$, $\langle m_z^2 \rangle_{e_g} = 1.04\mu_B^2$ and the rest, which is due to the mixed t_{2g}/e_g contribution. The fact that $\langle m_z^2 \rangle_{t_{2g}} \sim 1\mu_B^2$ is due to the low-spin d^5 configuration ($t_{2g}^3 \uparrow t_{2g}^2 \downarrow$) of Fe^{3+} ions. It is more interesting to discuss how so large contribution from the e_g orbitals appears.

The answer is that this moment is not due to Fe electrons, but actually it comes from oxygen holes. Due to a rather large oxidation state of Fe some of the electrons move from oxygen to iron (and create holes on oxygen sites; for the review see[15,16]). In a conventional DFT calculation this is a well known effect, which results in decrease of the local magnetic moment (the so-called decrease of the moment due to hybridization or covalency). But in paramagnetic DMFT we calculate not an ordered, but fluctuating moment, which is then squared and averaged over all QMC sweeps. Each time an electron comes from an oxygen it can have the same spin as Fe electrons (which are in d^5 low-spin configuration), or can have an opposite spin. Intra-atomic Hund's rule dictates the same spin configuration. On each QMC step in the impurity problem we have contributions both from the t_{2g} and e_g orbitals to the total $\langle m_z^2 \rangle$. The total magnetic moment is zero, since the spin moment in paramagnetic DMFT is fluctuating, but $\langle m_z^2 \rangle$ is not. Due to intra-atomic Hund's exchange in paramagnetic DMFT oxygen electrons will increase $\langle m_z^2 \rangle$ (not the total moment!) in a strong contrast to the DFT, where hybridization leads to decrease of the local moment. Moreover, since $t_{2g}^{\uparrow}$ states are completely filled in the case of Fe^{3+} and $pd\sigma$ bonding is much stronger than $pd\pi$ this contribution will largely affect $\langle m_z^2 \rangle_{eg}$.

The uniform magnetic susceptibility, $\chi_u(T)$, was calculated as a response to small external field (corresponding to Zeeman splitting of 0.01 eV) in DFT+DMFT and presented in Fig. 3 of the main text. In order to identify the origin of its nonmonotonic temperature behavior we performed an analysis of the spin susceptibility $\chi_{p-h}(T)$ employing the particle-hole bubble approximation (see e,g. Ref.[17]):

$$\chi_{p-h}(T) = -k_{\mathrm{B}} T \mathrm{Tr} \sum_{\mathbf{k}, i\omega_n} \hat{G}(\mathbf{k}, i\omega_n) \hat{G}(\mathbf{k}, i\omega_n). \tag{2}$$

Here $\hat{G}(\mathbf{k}, i\omega_n) = [(i\omega_n + \mu)\hat{I} - \hat{H}_{\mathrm{DFT}}(\mathbf{k}) + \hat{\Sigma}(i\omega_n)]^{-1}$ is the lattice Green's function, $i\omega_n$ are the fermionic Matsubara frequencies, μ is the chemical potential, $\hat{I}$ is the identity operator and $\hat{\Sigma}(i\omega_n)$ is the local self-energy. The operator $\hat{H}_{\mathrm{DFT}}(\mathbf{k})$ denotes the effective Hamiltonian computed by projection onto a set of Wannier functions with symmetry of p and d states. Spin susceptibility without the account of electronic correlations i.e. corresponding to DFT is obtained by setting $\hat{\Sigma}(i\omega_n) = \hat{0}$.

Temperature dependence of $\chi_{p-h}(T)$ corresponding to DFT and DMFT solutions is presented in Fig. S3 (left panel). First of all, one may see that $\chi_{p-h}^{\mathrm{DMFT}}(T)$ computed with DFT+DMFT single-particle Green functions basically preserves all features of the temperature evolution of $\chi_u(T)$ obtained in direct DFT+DMFT calculation. Namely, it demonstrates a decrease in the low-temperature region followed by a minimum and a quasi-linear increase in the region of higher temperatures. Secondly, we found that the particle-hole bubble $\chi_{p-h}^{\mathrm{DFT}}(T)$ computed within non-interacting Green functions ($\hat{\Sigma}(i\omega_n) = \hat{0}$) is strongly influenced by small variations of the chemical potential due to temperature broadening (Fig. S3 (right panel)). Specifically,

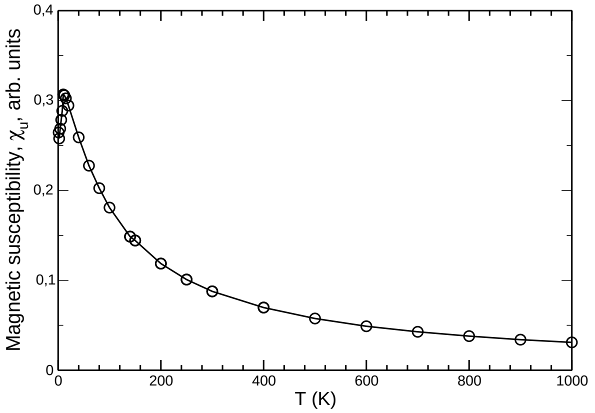

Figure S4. Magnetic susceptibility for FeO_2 with 25% of Fe vacancies obtained using QMC simulations[11] for classical Heisenberg model (the cluster size was chosen to be $L = 14$).

temperature dependence of $\chi_{p-h}^{\mathrm{DFT}}(T)$ obtained using the fixed chemical potential corresponding to $T = 0$ K does not reproduce the low-temperature behavior of $\chi_{p-h}^{\mathrm{DMFT}}(T)$. At the same time $\chi_{p-h}^{\mathrm{DFT}}(T)$ computed with temperature-dependent μ is qualitatively similar to $\chi_{p-h}^{\mathrm{DMFT}}(T)$. These observations along with the fact that electronic correlations do not cause a serious transformation of the energy spectrum allow to suggest that single-particle properties, in particular peculiarities of the single-particle density of states in the vicinity of the Fermi play a dominant role in the formation of the non-monotonic behavior of the uniform susceptibility.

This situation is thoroughly discussed in literature. In particular, it was mentioned that there is indeed an important contribution to the magnetic susceptibility in metals related to the temperature dependence of chemical potential[18]. Also, it was shown that thermal excitations of the low-energy states of the energy spectrum forming sharp peaks below the Fermi level can be responsible for similar anomalies of the uniform susceptibility in iron-based superconductors. Therefore, we conclude that anomalies of the temperature behavior of magnetic susceptibility the FeO_2 are most likely to be defined by its low-energy band structure. The quasi-linear growth of $\chi_u(T)$ for $T > T^* \sim 750$ K is then due to a strong peak in the DFT density of states centered at ~ -0.2 eV.

References

1. Blaha, P., Schwarz, K., Madsen, G., Kvasnicka, D. & Luitz, J. *WIEN2k, An Augmented Plane Wave + Local Orbitals Program for Calculating Crystal Properties* (Techn. Universität Wien, Wien, 2001).
2. Giannozzi, P. *et al.* QUANTUM ESPRESSO: a modular and open-source software project for quantum simulations of materials. *J. Physics: Condens. Matter* **21**, 395502 (2009). DOI 10.1088/0953-8984/21/39/395502.
3. Stevens, E. D., DeLucia, M. L. & Coppens, P. Experimental observation of the effect of crystal field splitting on the electron density distribution of iron pyrite. *Inorg. Chem.* **19**, 813–820 (1980). URL http://dx.doi.org/10.1021/ic50206a006. DOI 10.1021/ic50206a006.
4. Hu, Q. *et al.* FeO2 and FeOOH under deep lower-mantle conditions and Earth's oxygen–hydrogen cycles. *Nat.* **534**, 241–244 (2016). URL http://dx.doi.org/10.1038/nature18018. DOI 10.1038/nature18018.
5. Anisimov, V. I. *et al.* Coulomb repulsion and correlation strength in LaFeAsO from density functional and dynamical mean-field theories. *J. Physics: Condens. Matter* **21**, 075602 (2009). URL http://stacks.iop.org/0953-8984/21/i=7/a=075602?key=crossref.1d347f13f2ba986e0606d11359b0165d. DOI 10.1088/0953-8984/21/7/075602.
6. Anisimov, V. I., Poteryaev, A. I., Korotin, M. A., Anokhin, A. O. & Kotliar, G. First-principles calculations of the electronic structure and spectra of strongly correlated systems: dynamical mean-field theory. *J. Physics: Condens. Matter* **9**, 7359 (1997). URL http://iopscience.iop.org/0953-8984/9/35/010.
7. Poteryaev, A. *et al.* AMULET. URL http://amulet-code.org.

8. Dyachenko, A. A., Shorikov, A. O., Lukoyanov, A. V. & Anisimov, V. I. Two successive spin transitions in a wide range of pressure and coexistence of high- and low-spin states in clinoferrosilite FeSiO3. *Phys. Rev. B* **93**, 245121 (2016). DOI 10.1103/PhysRevB.93.245121.

9. Gull, E., Millis, A. J., Lichtenstein, A. I., Troyer, M. & Werner, P. Continuous-time Monte Carlo methods for quantum impurity models. *Rev. Mod. Phys.* **83** (2011). DOI 10.1103/RevModPhys.83.349.

10. Jang, B. G., Kim, D. Y. & Shim, J. H. Metal-insulator transition and the role of electron correlation in FeO 2. *Phys. Rev. B* **95**, 075144 (2017). DOI 10.1103/PhysRevB.95.075144.

11. Bauer, B. *et al.* The ALPS project release 2.0: open source software for strongly correlated systems. *J. Stat. Mech. Theory Exp.* 05001 (2011). URL http://iopscience.iop.org/1742-5468/2011/05/P05001.

12. Korotin, D. *et al.* Construction and solution of a Wannier-functions based Hamiltonian in the pseudopotential plane-wave framework for strongly correlated materials. *The Eur. Phys. J. B* **65**, 91 (2008). arXiv:0801.3500v4.

13. Liechtenstein, A. I., Anisimov, V. I. & Zaanen, J. Density-functional theory and strong interactions: Orbital ordering in Mott-Hubbard insulators. *Phys. Rev. B* **52**, 5467–5471 (1995). URL http://prola.aps.org/abstract/PRB/v52/i8/pR5467{_}1.

14. Silver, R., Sivia, D. & Gubernatis, J. Maximum-entropy method for analytic continuation of quantum Monte Carlo data. *Phys. Rev. B* **41**, 2380 (1990).

15. Khomskii, D. Unusual valence, negative charge-transfer gaps and self-doping in transition-metal compounds. *Lith. J. Phys.* **37**, 65 (1997).

16. Sawatzky, G., Green, R., Mall, E., Bc, V. & Vt, C. The Explicit Role of Anion States in High-Valence Metal Oxides. In Pavarini, E., Koch, E., van den Brink, J. & Sawatzky, G. (eds.) *Quantum Materials: Experiments and Theory Modeling and Simulation*, vol. 6, 1–36 (Verlag des Forschungszentrum, Jülich, 2016).

17. Georges, A., Krauth, W. & Rozenberg, M. J. Dynamical mean-field theory of strongly correlated fermion systems and the limit of infinite dimensions. *Rev. Mod. Phys.* **68**, 13–125 (1996). URL http://link.aps.org/doi/10.1103/RevModPhys.68.13. DOI 10.1103/RevModPhys.68.13.

18. Irkhin, V. Y. & Irkhin, Y. P. *Electronic Structure, Correlation Effects and Physical Properties of D- and F-metals and Their Compounds* (Cambridge International Science Publishing, Limited, 2007). URL https://books.google.ru/books?id=R5Qr{_}dxCbg4C.
